# Ethanol mediates the interaction between *Caenorhabditis elegans* and the nematophagous fungus *Purpureocillium lavendulum*

**DOI:** 10.1128/spectrum.01270-23

**Published:** 2023-08-10

**Authors:** Xue-Mei Zhuang, Zhi-Yi Guo, Meng Zhang, Yong-Hong Chen, Feng-Na Qi, Ren-Qiao Wang, Ling Zhang, Pei-Ji Zhao, Chao-Jun Lu, Cheng-Gang Zou, Yi-Cheng Ma, Jianping Xu, Ke-Qin Zhang, Yan-Ru Cao, Lian-Ming Liang

**Affiliations:** 1 State Key Laboratory for Conservation and Utilization of Bio-Resources in Yunnan and The Key Laboratory for Southwest Microbial Diversity of the Ministry of Education, Yunnan University, Kunming, China; 2 Department of Biology, McMaster University, Hamilton, Ontario, Canada; 3 College of Agriculture and Life Sciences, Kunming University, Kunming, China; Chinese Academy of Sciences, Shanghai, China

**Keywords:** nematophagous fungi, fungal infection, innate immunity, ethanol, ethanol dehydrogenase, pathogen recognition

## Abstract

Accurately recognizing pathogens by the host is vital for initiating appropriate immune response against infecting microorganisms. *Caenorhabditis elegans* has no known receptor to recognize pathogen-associated molecular pattern. However, recent studies showed that nematodes have a strong specificity for transcriptomes infected by different pathogens, indicating that they can identify different pathogenic microorganisms. However, the mechanism(s) for such specificity remains largely unknown. In this study, we showed that the nematophagous fungus *Purpureocillium lavendulum* can infect the intestinal tract of the nematode *C. elegans* and the infection led to the accumulation of reactive oxygen species (ROS) in the infected intestinal tract, which suppressed fungal growth. Co-transcriptional analysis revealed that fungal genes related to anaerobic respiration and ethanol production were up-regulated during infection. Meanwhile, the ethanol dehydrogenase Sodh-1 in *C. elegans* was also up-regulated. Together, these results suggested that the infecting fungi encounter hypoxia stress in the nematode gut and that ethanol may play a role in the host–pathogen interaction. Ethanol production *in vitro* during fungal cultivation in hypoxia conditions was confirmed by gas chromatography-mass spectrometry. Direct treatment of *C. elegans* with ethanol elevated the *sodh-1* expression and ROS accumulation while repressing a series of immunity genes that were also repressed during fungal infection. Mutation of *sodh-1* in *C. elegans* blocked ROS accumulation and increased the nematode’s susceptibility to fungal infection. Our study revealed a new recognition and antifungal mechanism in *C. elegans*. The novel mechanism of ethanol-mediated interaction between the fungus and nematode provides new insights into fungal pathogenesis and for developing alternative biocontrol of pathogenic nematodes by nematophagous fungi.

**IMPORTANCE:**

Nematodes are among the most abundant animals on our planet. Many of them are parasites in animals and plants and cause human and animal health problems as well as agricultural losses. Studying the interaction of nematodes and their microbial pathogens is of great importance for the biocontrol of animal and plant parasitic nematodes. In this study, we found that the model nematode *Caenorhabditis elegans* can recognize its fungal pathogen, the nematophagous fungus *Purpureocillium lavendulum*, through fungal-produced ethanol. Then the nematode elevated the reactive oxygen species production in the gut to inhibit fungal growth in an ethanol dehydrogenase-dependent manner. With this mechanism, novel biocontrol strategies may be developed targeting the ethanol receptor or metabolic pathway of nematodes. Meanwhile, as a volatile organic compound, ethanol should be taken seriously as a vector molecule in the microbial–host interaction in nature.

## INTRODUCTION

Nematodes are diverse and broadly distributed across many ecosystems on earth (1 – 3). Many nematodes are parasites capable of causing significant damages to the health of plants, animals, and humans (4, 5). Biological control of parasitic nematodes with their natural microbial enemies is an environmentally friendly approach and has attracted increasing attention for studying the mechanisms of nematode–microbe interactions. *Caenorhabditis elegans*, a free-living soil nematode species commonly exposed to many environmental microorganisms, including food microbes and pathogens, is a common model organism for studying animal–microbe interactions (6 – 9). Several human pathogenic microbes, such as *Pseudomonas aeruginosa* and *Salmonella enterica*, can also infect the intestinal *C. elegans* (10, 11). Indeed, *C. elegans* is commonly used as a model host for studying host–pathogen interaction of human pathogens (12). These studies have demonstrated that *C. elegans* can defend against pathogenic microbes by relying on the defense mechanisms of its epithelial cells through various innate immune pathways, such as mitogen-activated protein kinases (P38 MAPK signaling pathway, NSY-1/SEK-1/PMK-1) (13 – 15), Daf-2/Daf-16 pathway (16, 17), and the RNA interference (RNAi) machinery, which is important for antiviral defense (18). Accurately identifying pathogens by the host is key to initiating the immune response against pathogen infestation. Innate immune cells such as macrophages and dendritic cells detect molecular components of foreign microorganisms known as pathogen-associated molecular patterns (PAMPs) through pattern-recognition receptors (PRRs), which include three major families—Toll-like receptors (TLRs), RIG-I-like receptors (RLRs), and NOD-like receptors (NLRs). These receptors recognize a wide range of proteins, nucleic acids, lipids, and carbohydrates derived from foreign microorganisms (19). As a structurally and genetically simple animal, *C. elegans* lacks some of the constituent elements of PRRs, such as not having NFκB homologous proteins and NLRs, and its only TLR has no role in natural immunity and does not respond to microbe-associated molecular patterns (20). Recently, RLRs were characterized as functional in mediating the intracellular pathogen response upon viral infection in *C. elegans* (21). In response to both intestinal and epidermal infection, epithelial cells of *C. elegans* up-regulate secreted antimicrobial peptides, detoxifying enzymes, and efflux pumps, with distinct responses to distinct pathogens (22). These studies indicate that they can differentiate different pathogens. However, almost nothing is known about what the mechanisms might be.

*Purpureocillium lavendulum* (previously mischaracterized as *Paecilomyces lilacinus*) is a typical soil and plant root symbiotic fungus and a common biocontrol fungus against parasitic nematodes (23, 24). Our previous work found that *C. elegans* can ingest conidia of *P. lavendulum*, which can germinate in the worm’s gut, then kill and degrade the nematode (25). In this paper, we report our recent studies using this pathogen–host model to study their interactions. We found that the small molecule, ethanol, produced by the fungal pathogen, can be recognized by *C. elegans* and induce defense against fungal infection.

## RESULTS

### Transcriptome analysis revealed ethanol metabolic genes involved in fungal–nematode interactions

To gain an overall view of the gene expression of the fungus and the nematode during fungal infection of *C. elegans*, a transcriptomic analysis was carried out. Twenty-four-hour and 5-day post-infection samples were collected, and RNAs of both the nematode and the fungus were extracted and sequenced. Uninfected nematodes and pure culture-grown fungi were used as controls.

In the 24-h infection sample, there were 6,742 genes differently expressed between infected and uninfected *C. elegans*, of which 2,769 were up-regulated and 3,973 were down-regulated. Gene ontology (GO) enrichment analysis showed that innate immune response and mitotic cell cycle-related biological process (BP) terms were enriched for the differentially expressed genes (DEGs). It is worth noting that many immune-related processes were down-regulated. The Kyoto Encyclopedia of Genes and Genomes (KEGG) enrichment analysis showed that autophagy-animal, fatty acid metabolism, amino acid metabolism, and longevity regulating pathway—worm pathways—were enriched for the DEGs (Fig. S1A and B).

In the 5-day infected nematode samples, there are 2,462 DEGs, of which 1,318 were up-regulated and 1,144 were down-regulated. GO enrichment analysis showed that chromatin remodeling, reproduction, neuropeptide signaling, DNA repair-related BP terms, etc., were enriched for the DEGs. KEGG enrichment analysis showed that spliceosome, Fanconi anemia, homologous recombination, and oxidative phosphorylation pathways were enriched for the DEGs (Fig. S1C and D). In the 5-dayinfected samples, a total of 2,885 genes were differentially expressed in the fungi, of which 948 were up-regulated and 1,937 were down-regulated. GO terms such as purine ribonucleotide metabolic process, ATP metabolic process, and ribose phosphate metabolic process were enriched (Fig. S2). Only the ribosome pathway was enriched for the KEGG enrichment analysis.

**Fig 1:**
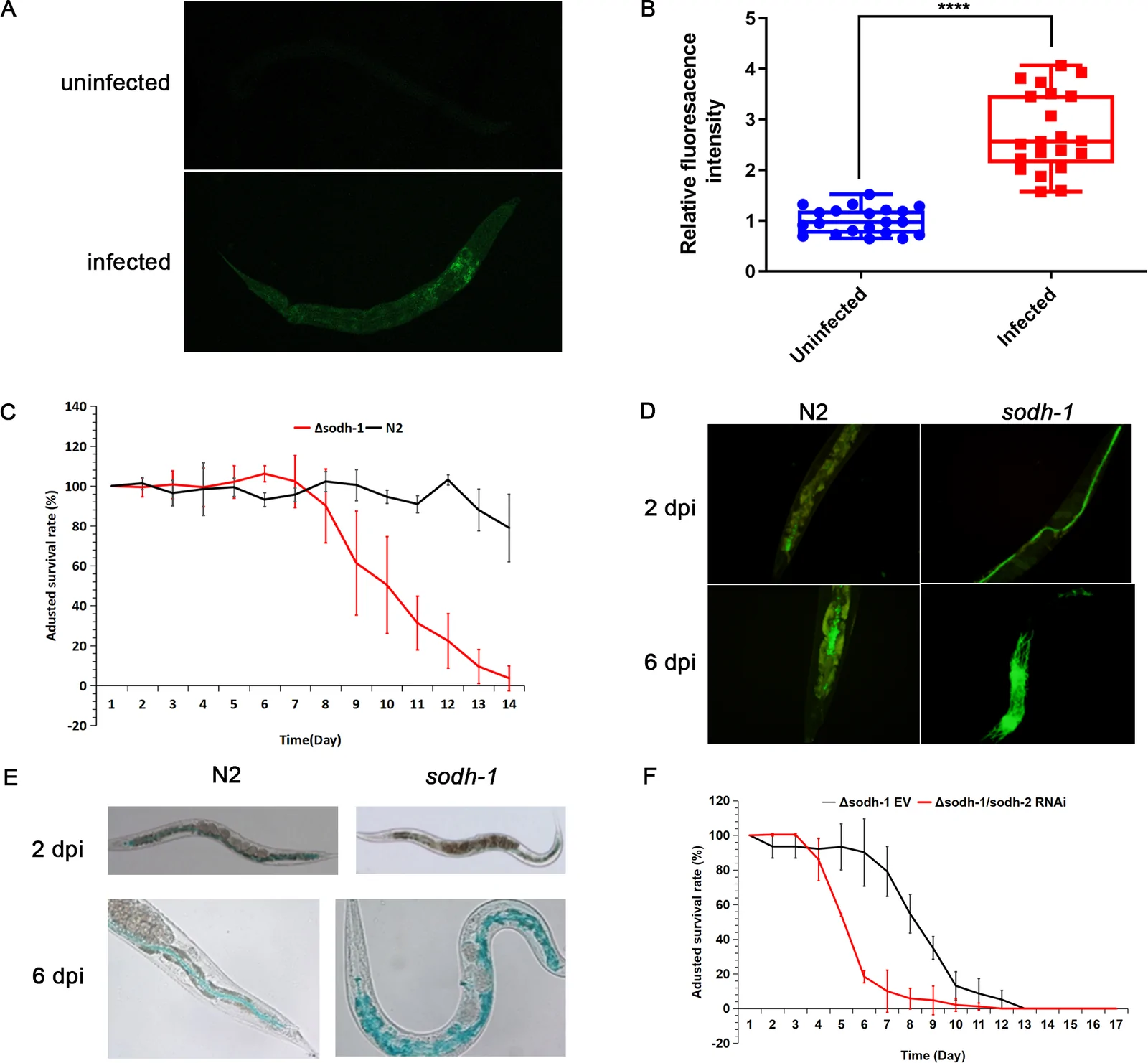
Hypoxia levels in *C. elegans* and the impact of *sodh-1* and *sodh-2* mutants on *P. lavendulum* infection. (**A**) Comparison of hypoxia levels in *C. elegans* before and after fungal infection using fluorescence staining (Image-iT Green). (**B**) Quantify and compare the fluorescence values between infected (*n* = 21) and uninfected (*n* = 21) nematodes. (**C**) Comparison of adjusted survival rates between *sodh-1* mutant strains and N2 strains infected by *P. lavendulum*. (**D**) Germination and growth of green fluorescent protein-labeled *P. lavendulum* in *sodh-1* mutant and wild-type strains. (**E**) Intestinal integrity of *sodh-1* mutant strains and wild-type strains was compared when infected by food blue staining. (**F**) Comparison of the survival rates of *sodh-2* RNAi and EV, the empty RNAi vector plasmid (control, of *C. elegans* strain *sodh-1* exposed to *P. lavendulum*.

Intriguingly, we found that the ethanol dehydrogenase *sodh-1* of *C. elegans* was up-regulated by 3.5 folds in a 24-h infection. In the 5-day infection samples, the expression level of the pyruvate decarboxylase gene *pdc1* of *P. lavendulum* was increased by 7.3 times, and the alcohol dehydrogenase gene adh1 was increased by 5.6 times in the mycelia growing inside *C. elegans*. Ethanol dehydrogenase *adh1* and pyruvate decarboxylase *pdc1* are two critical genes for ethanol production in eukaryotic anaerobic respiration (26 – 29). In addition, two orthologs of human *hyou1* (hypoxia up-regulated 1), *t24h7.2* and *t14g8.3*, were found up-regulated in the *C. elegans* transcriptome and may also play a role in the hypoxia response of *C. elegans*. To further characterize the oxygen levels in the midgut of *C. elegans* after infection, we stained the midgut with the Image-iT Green Hypoxia Reagent (ThermoFisher Scientific, Foster City, CA, USA) and observed a significant decrease in oxygen levels in the midgut of infected worms compared to uninfected controls (Fig. 1A and B). This result suggests that *P. lavendulum* may encounter hypoxia during its germination and growth in the intestinal tract of *C. elegans* and that the nematode likely responded by up-regulating the expression level of the ethanol dehydrogenases. However, it is not clear whether the putative ethanol-mediated interaction plays a role in the infection and defense processes of the pathogens and hosts.

### Ethanol dehydrogenase in *C. elegans* is involved in the defense against fungal infection

To test our hypothesis that sodh-1 was involved in the worm’s defense against *P. lavendulum*, we carried out a bioassay on wild-type *C. elegans* (N2) and *sodh-1* mutant strains. We found that when infected by *P. lavendulum, sodh-1* mutant *C. elegans* had a significantly higher mortality rate than the N2 strain (Fig. 1C). To investigate the infection process of the fungus in the nematode intestine tract, we used the green fluorescent protein (GFP)-labeled *P. lavendulum* conidia to infect the nematodes, as previously reported (25). It was observed that the conidia germination of *P. lavendulum* in the intestinal tract of the *sodh-1* mutant was faster than in the wild-type worms (Fig. 1D). Meanwhile, the intestinal injury rate of *sodh-1* mutant nematode was also higher than that of wild-type after infection with *P. lavendulum*, as indicated by the Food Brilliant Blue staining (Fig. 1E). The results indicate that ethanol dehydrogenase *sodh-1* is involved in the defensing against *P. lavendulum* infection.

*Sodh-2* encodes another alcohol dehydrogenase. To test the role of *sodh-2* in resisting *P. lavendulum* infection, bioassays were carried out on *sodh-2* RNAi nematodes. As shown in Fig. S3, no significant difference in survival rates was detected between *sodh-2* RNAi nematodes and the control (N2 EV). Then the *sodh-1* mutant worm was subjected to *sodh-2* RNAi and infected by *P. lavendulum*. The Δ*sodh-1*/*sodh-2* RNAi nematodes were more susceptible to *P. lavendulum* infection than the *sodh-1* ko worms (Fig. 1F). The results suggest that *sodh-2* likely plays a redundant role in the natural immunity of *C. elegans* against *P. lavendulum* infection.

### *sodh-1* function in innate defense against fungal infection depended on ROS

**Fig 2:**
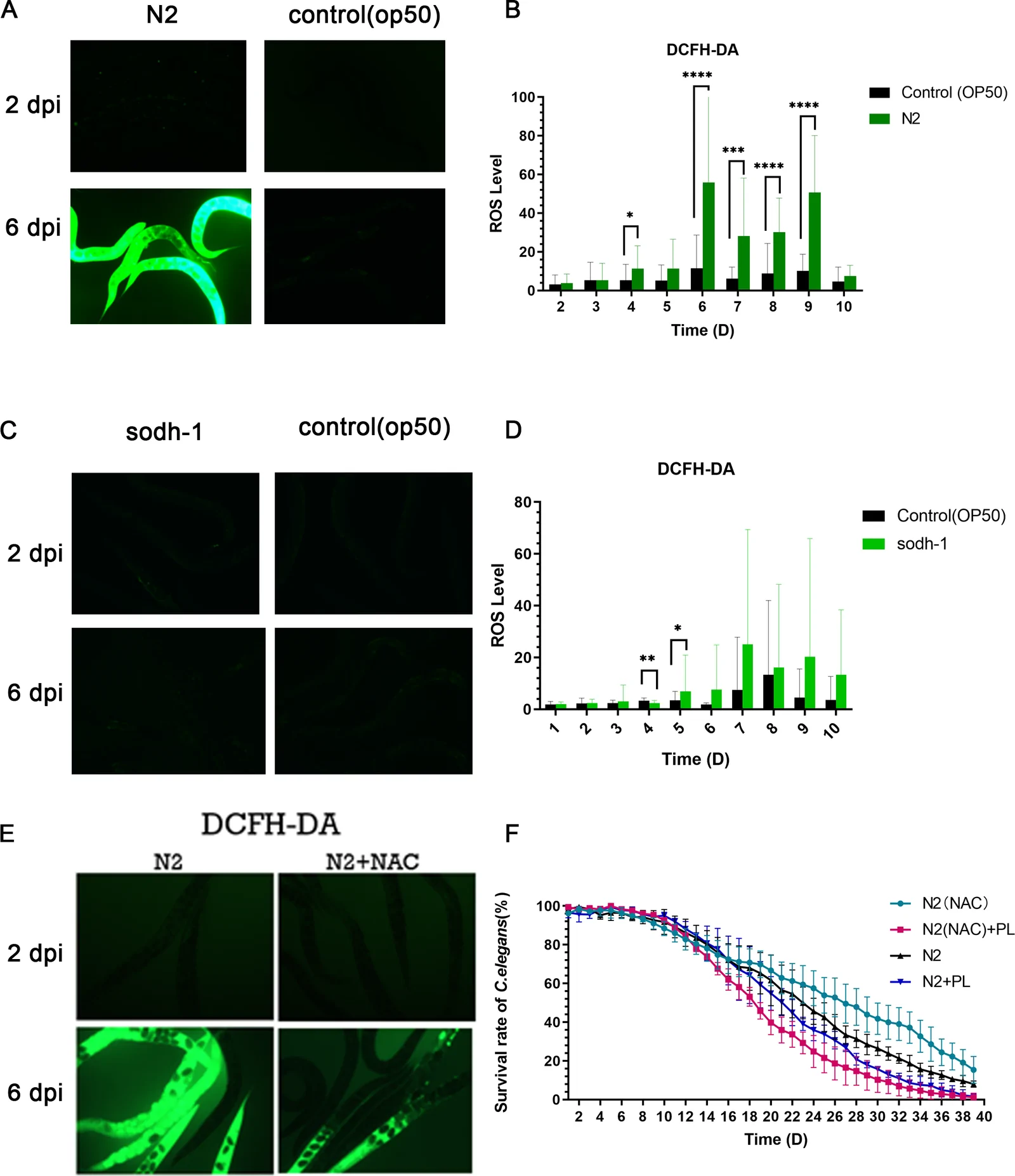
ROS accumulated in fungal-infected nematodes. (**A**) ROS staining in *C. elegans* (**N2**) infected or uninfected by *P. lavendulum*. (**B**) Statistical analysis of panel **A**. (**C**) ROS staining in *C. elegans* (*sodh-1*) infected or uninfected by *P. lavendulum*. (**D**) Statistical analysis of panel **C**. (**E**) *C. elegans* (**N2**) infected by *P. lavendulum*. (**F**) Comparison of survival rates between NAC-treated nematodes and untreated nematodes infected by *P. lavendulum*.

Reactive oxygen species (ROS) are a group of chemically defined active molecules derived from molecular oxygen (30), produced primarily as a by-product of the mitochondria and other cellular components (31). ROS and antioxidant compounds are also important signaling molecules in the oxidative stress response (32, 33). The functions of ROS in *C. elegans* innate immunity include directly inhibiting the growth of pathogenic microorganisms (34, 35) or as a signal molecule to activate the downstream genes of the P38 MAPK pathway (NSY-1/SEK-1/PMK-1) to activate SKN-1 to initiate innate immunity (36, 37). We stained the intestine with the fluorescent probe, dihydroethidium (DHE), of ROS. We found that *P. lavendulum* infection with N2 nematodes for 6 days led to intestinal ROS accumulation, but infection of *sodh-1* mutant nematodes by *P. lavendulum* did not cause the accumulation of intestinal ROS (Fig. 2A through D). *N*-acetyl-L-cysteine (NAC) is an antioxidant containing the sulfhydryl group. When NAC was added during infection, there was less ROS accumulation (Fig. 2E), and the nematodes were more susceptible than those not treated by NAC (Fig. 2F). The germination and growth of the conidia in nematodes treated with NAC were more vigorous than in nematodes not treated with NAC (Fig. S4A). The body-cavity leakage of nematodes on day 6 was more significant than that of the control group (Fig. S4B). Therefore, we speculate that ROS production depends on *sodh-1* and they play an essential role in defending against fungal infection.

### Anaerobic respiration and ethanol synthases in *P. lavendulum* are required for full virulence during nematode infection

**Fig 3:**
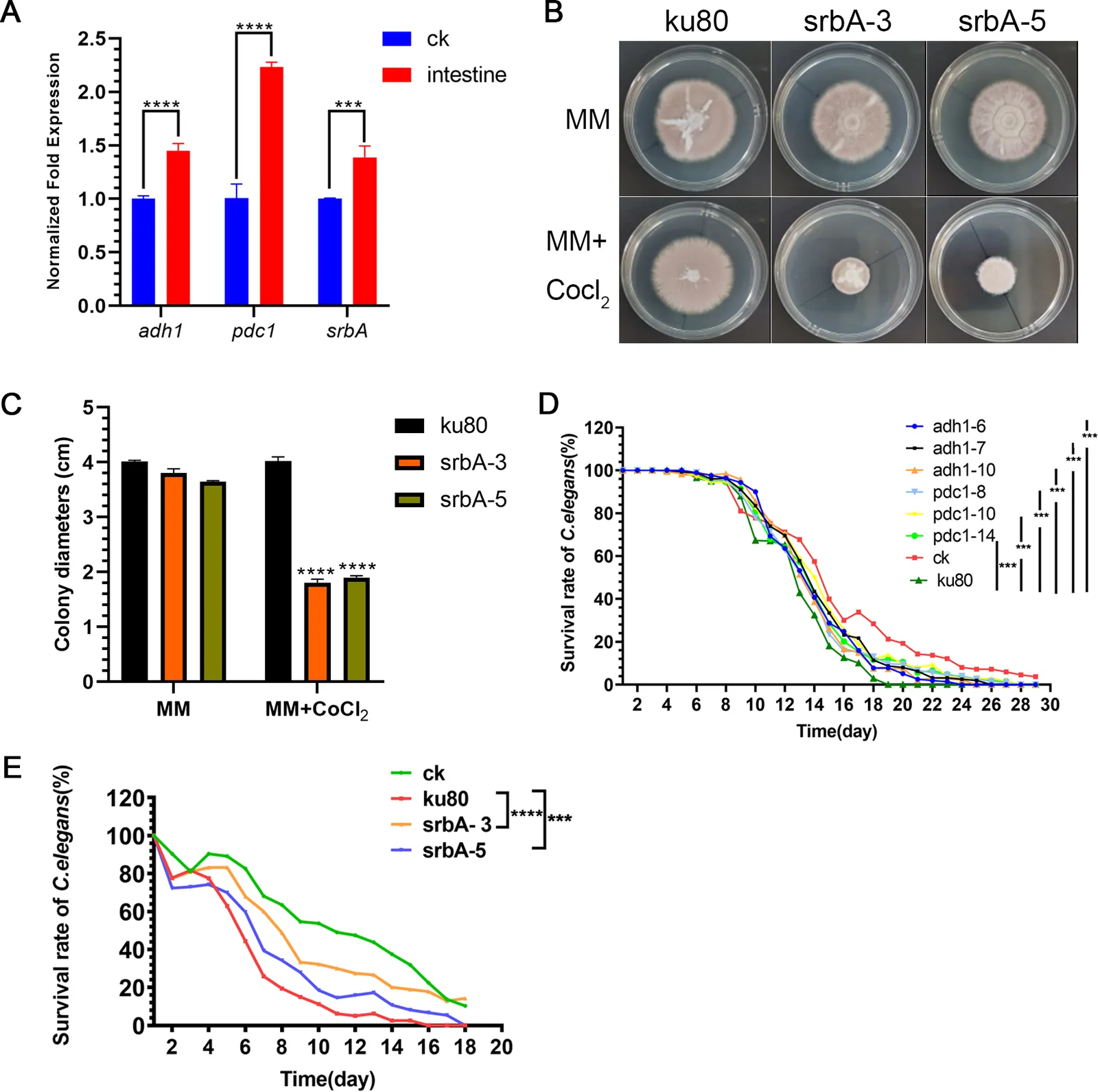
Knockout of anaerobic respiration-related genes affects hypoxia tolerance and fungal pathogenesis. (**A**) Comparison of the expression levels of *adh-1*, *pdc-1*, and *srbA* in fungal hyphae cultured *in vitro* (ck) and those invading and growing inside nematode hosts (intestine) by real-time quantitative PCR. (**B**) Colony morphology of *srbA* knockout strains and the *ku80* strain on MM medium with and without CoCl_2_. (**C**) Colony diameter statistics of the three strains. (**D**) Comparison of survival rates between *adh-1*, *pdc-1,* and the *ku80* strain-infected nematodes. (**E**) Comparison of survival rates between *srbA* mutants and the *ku80* strain-infected nematodes. ***, *P* < 0.001; ****, *P* < 0.0001.

Oxygen availability affects cell differentiation, survival, and function and profoundly impacts tissue homeostasis, inflammation, and immunity (38). The amount of oxygen available to eukaryotic cells is critical for determining overall cell metabolism (39 – 41). Hypoxia has a significant effect on controlling the expression of proteins involved in various BPs (42). Pyruvate decarboxylase PDC1 and alcohol dehydrogenase ADH1 are two essential enzymes of the ethanol fermentation pathway in eukaryotes. However, the roles of ethanol dehydrogenase and pyruvate decarboxylase on the growth and virulence of *P. lavendulum* under low oxygen are still not clear. The sterol regulatory element-binding protein SrbA belongs to the basic helix–loop–helix transcription factor family and plays a key role in anti-fungal resistance and virulence (43). In *Aspergillus fumigatus*, researchers also found that the loss of *SrbA* resulted in the inability of fungal mutants to grow in a low-oxygen environment (43 – 45). To validate the results of transcriptome analysis, we performed real-time qPCR on the three genes, *adh1*, *pdc1,* and *srbA*, and the results showed that all of them were significantly up-regulated in the intestine of infected nematodes (Fig. 3A). We then knocked out the three genes with a homologous recombination strategy. A diagram showing gene knockout and transformant confirmation is presented in Fig. S5. The srbA mutants showed much more sensitivity to hypoxia than the *ku80* strain when cultured on minimal medium (MM) with CoCl_2_ (for simulation of a hypoxia environment) (Fig. 3B and C). No significant difference was found between the ku80 strain and the *adh1* and *pdc1* mutants. In addition, the three knockout strains showed significantly lower nematicidal activity than that of the ku80 strain (Fig. 3D and E).

Overall, our bioassay results showed that, compared to the wild-type strain, the nematicidal activities of several fungal knockout strains were significantly reduced. These results suggest that these three genes are required for full virulence of *P. lavendulum* in infecting *C. elegans*. Moreover, the functions of the three genes are all related to the hypoxia regulation of *P. lavendulum*, which further confirms our previous speculation that *P. lavendulum* may encounter hypoxia stress in the intestinal tract of *C. elegans*.

### *P. lavendulum* produced ethanol under hypoxia condition

**Fig 4:**
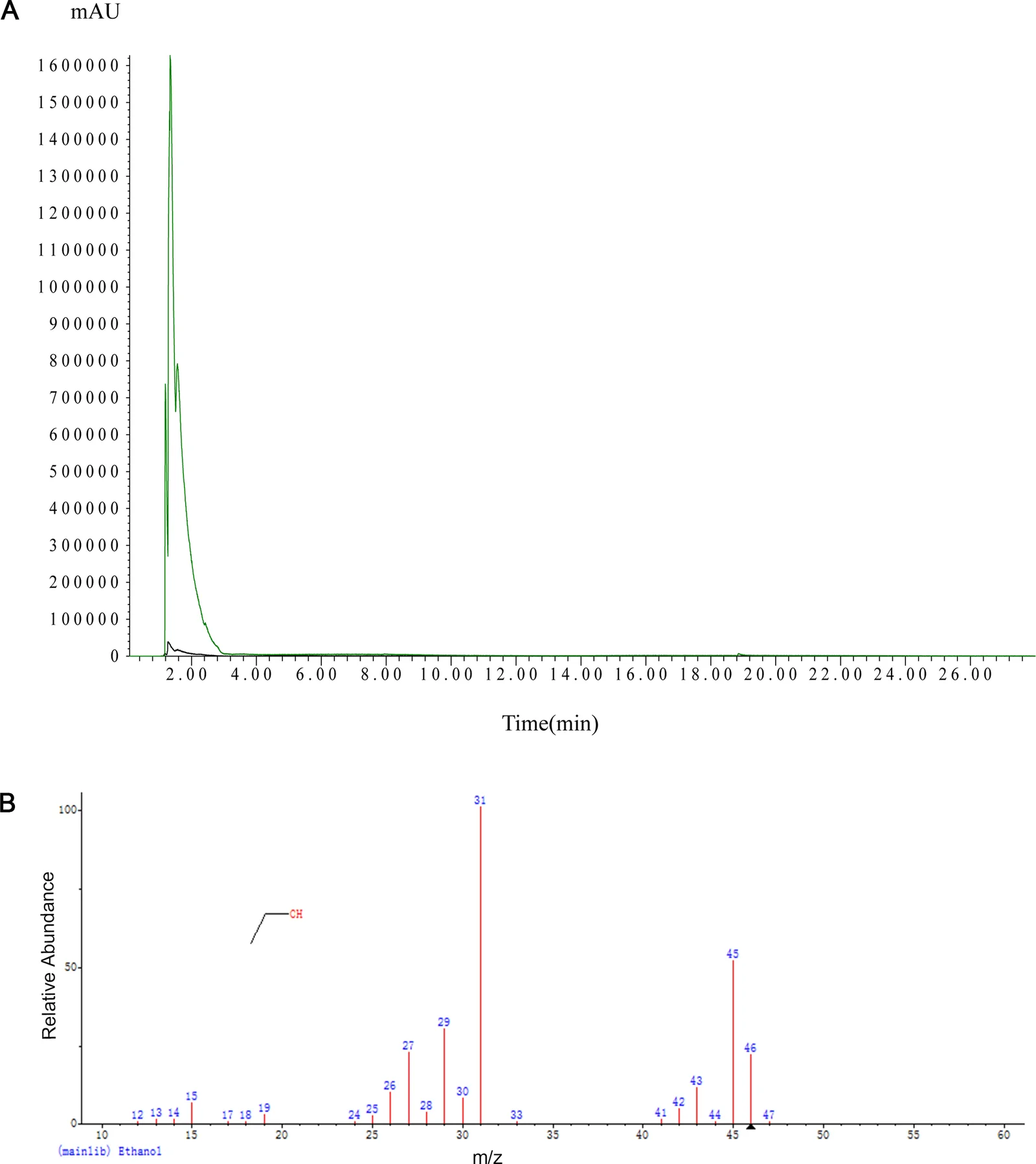
Detection of ethanol production by GC-MS. (**A**) Chromatogram of ethanol produced by *P. lavendulum* under hypoxic culture (black peak) and control (pure ethanol, green peak). (**B**) Mass spectrogram of ethanol.

In the absence of oxygen, organisms derive energy from the degradation and metabolism of sugars, in which pyruvate is catalyzed by pyruvate decarboxylase (*pdc1*) to lose carbon dioxide and produce acetaldehyde, which is then reduced to ethanol by ethanol dehydrogenase 1 (*adh1*) (46, 47). In the interactive transcriptome of *P. lavendulum* infection in *C. elegans*, alcohol dehydrogenase and pyruvate decarboxylase of *P. lavendulum*, two essential enzymes in anaerobic respiration producing ethanol, were both significantly increased. Thus, we speculated that *P. lavendulum* might encounter hypoxia stress in nematode intestines and produce ethanol. Through a literature review, it is found that solid-phase microextraction can extract volatile trace compounds like ethanol (48 – 51). Therefore, we put microaerobic airbags in a sealed tank to create a relatively low-oxygen environment. At the same time, 10 mL of MM liquid culture medium with and without the inoculation of *P. lavendulum* ku80 was put into the sealed tank under the exact condition of regular oxygen, at 28°C, 180 rpm for 10 days. Then, a solid-phase microextraction device was used to heat at 55°C and extract the fungal culture medium in a low-oxygen and normoxic environment for 12 h to obtain the volatile compounds produced by the strain. Finally, gas chromatography-mass spectrometry (GC-MS) was used to detect the results. As shown in Fig. 4, we found that *P. lavendulum* could produce ethanol through anaerobic respiration under hypoxic conditions, while it could not produce ethanol under normoxic conditions. The specific composition of volatile compounds in normoxic and hypoxic cultures is shown in Tables S2 and S3.

### Ethanol treatment-induced *sodh-1* expression and ROS accumulation in nematodes

**Fig 5:**
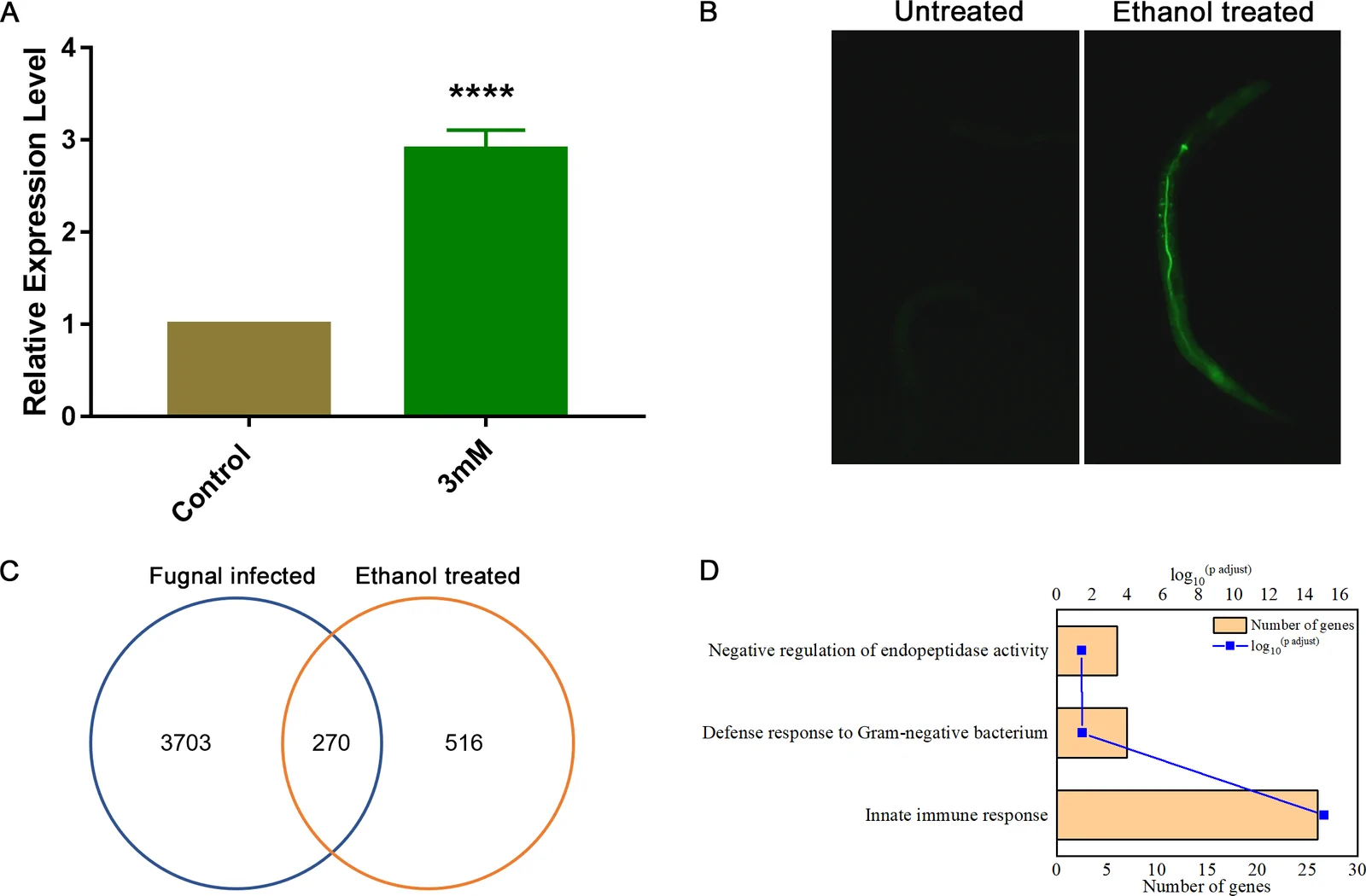
Ethanol treatment elevated *sodh-1* expression, ROS accumulation, and fungal-infection-related different gene expression in *C. elegans*. (**A**) Expression of *sodh-1* in nematodes treated with 3 mM ethanol; ****P < 0.0001. (**B**) The accumulation of ROS in the intestinal tract of nematodes was observed by dichloro-dihydro-fluorescein diacetate staining after ethanol treatment. (**C**) Venn diagram of gene numbers of fungal-infected (24 h) and ethanol-treated nematodes. (**D**) GO enrichment analysis of the 270 genes shared in panel **A**.

According to the above results, it is speculated that *P. lavendulum* might carry out anaerobic respiration and produce ethanol in the nematode intestines. To characterize the role of ethanol in the fungal–nematode interaction, the nematodes were treated with 3 mM/L ethanol for 8 h. As the alcohol dehydrogenase *sodh-1* is involved in nematode innate immunity against fungal infection, we tested the expression level of sodh-1 in nematode after ethanol treatment. Real-time PCR showed that the *sodh-1* gene was significantly up-regulated in nematodes treated with 3 mM ethanol for 8 h (Fig. 5A).

With dichloro-dihydro-fluorescein diacetate (DCFH-DA) staining, we found a significant accumulation of ROS in the intestinal tract of nematodes treated with ethanol compared with nematodes treated without ethanol (Fig. 5B). These results suggest that the presence of ethanol may stimulate the accumulation of ROS in nematodes. Furthermore, the results are consistent with our hypothesis that *P. lavendulum* produces ethanol in the intestinal tract of nematodes, and then ethanol stimulates the expression of nematode *sodh-1*, thus initiating the production of ROS.

### Transcriptome analysis of nematodes treated with ethanol

Based on the above results, nematodes directly treated with 3 mM ethanol showed the accumulation of intestinal ROS and the significant up-regulation of the ethanol dehydrogenase *sodh-1*. We then asked which aspects of nematodes reaction were induced. Transcriptome analyses of the third-instar nematodes treated with 3 mM ethanol for 8 h and those not treated with ethanol were compared using RNA-seq. A total of 1,328 genes were significantly differentially expressed in *C. elegans* treated with and without ethanol, of which 542 genes were up-regulated and 786 genes were down-regulated.

For the up-regulated genes, GO enrichment analysis showed enriched BPs including nucleoside bisphosphate metabolic-related processes, thioester metabolic processes, fatty acid biosynthetic and metabolic processes, sphingolipid biosynthetic and metabolic processes, and carboxylic acid biosynthetic and metabolic processes. KEGG enrichment analysis showed enrichment in longevity regulating pathway—worm, biosynthesis of unsaturated fatty acids, fatty acid degradation, PPAR signaling pathway, sphingolipid metabolism, etc. (Fig. S6).

GO enrichment analysis showed that the down-regulated genes were enriched in BP terms such as immune system process, innate immune response, immune response, response to external biotic stimulus, defense response to other organisms, and several other immunity-related BP terms. The expression of many lipases, heat shock proteins, C-type lectins, lysozymes, and F-box A proteins was down-regulated (Fig. S7A). KEGG enrichment analysis showed enrichment in lysosome, fatty acid degradation, PPAR signaling pathway, etc. (Fig. S7B). There were 270 genes down-regulated both in fungal-infected (24 h post-infection) and ethanol-treated *C. elegans*. GO enrichment analysis showed that genes were enriched in innated immune response, defense response to gram-negative bacterium, and negative regulation of endopeptidase activity (Fig. 5C and D). These results indicated that ethanol produced by fungi has an immune repression effect during the infection process. Therefore, the up-regulation of the alcohol dehydrogenase, *sodh-1*, not only detoxifies alcohol but also inhibits fungal growth by increasing ROS levels, thus compensating for the damage caused by ethanol inhibition of the nematode immune system.

## DISCUSSION

Activation of innate immunity involves the engagement of a set of germline-encoded “pattern recognition receptors,” which recognize common microbial structural moieties, such as lipopolysaccharide, from the cell walls of gram-negative bacteria (52). However, *C. elegans* lacks many of the cell surface and intracellular receptors involved in innate immunity in other species (53). Proteins related to pathogen recognition in *C. elegans* remain to be fully explored (54). Here we show that *C. elegans* can recognize the fungal produced ethanol, which may serve as a PAMP-like molecule in fungal infection.

In the first stage of fungal infection, the conidia were ingested into the intestinal tract of nematodes, and the germination and growth were inhibited by the hypoxia environment inside the worm. This hypoxia may be the main reason for the latent period of fungal infestation (at this stage, many conidia were excreted by nematodes). The transcriptome analysis and real-time PCR showed significant up-regulation of the pyruvate decarboxylase and ethanol dehydrogenase genes, which are homologous to those found in the brewer's yeast *Saccharomyces cerevisiae* and responsible for ethanol biosynthesis (55). Furthermore, the results of hypoxia staining in both infected and uninfected nematodes provided evidence to suggest that the fungi may carry out anaerobic respiration when infecting the gut of *C. elegans*. Therefore, both sets of results strongly support the notion that the fungi are capable of anaerobic respiration during gut infection. SrbA plays a crucial role in the adaptation of *Aspergillus fumigatus* to low oxygen conditions by regulating ergosterol biosynthesis, iron uptake, nitrate assimilation, and heme biosynthesis (43). This transcription factor plays a crucial role in regulating various BPs during adaptation to low oxygen, and thus knocking it out affects the growth of *P. lavendulum* on cobalt chloride-supplemented media. Anaerobic respiration is only a compensatory mechanism for adaptation to low oxygen, and it may require longer cultivation time or truly low oxygen conditions instead of cobalt chloride-simulated conditions to affect the growth of the *adh1* and *pdc1* knockout strains. The reduced virulence of the two knockout strains indicates that they also have reduced adaptability to the nematode gut.

Cultivation in the hypoxia environment led to ethanol production in *P. lavendulum*. Besides the brewer’s yeast, the filamentous fungal pathogen *A. fumigatus* has also been reported to produce ethanol in a hypoxia environment and may represent an adaptation strategy for living in an alveolar hypoxic microenvironment (56). Ethanol at high concentration is harmful for humans, especially in the liver. The pathogenesis of alcoholic liver disease is due to malnutrition as well as to ethanol’s hepatotoxicity linked to its metabolism by means of the alcohol dehydrogenase and cytochrome P450 2E1 (CYP2E1) pathways, resulting in the production of toxic acetaldehyde, changes in oxidation–reduction (redox) potentials, and oxidative stress (57). In the current study, we showed that ethanol treatment caused down-regulation of genes involved in immunity-related pathways, indicating a role for ethanol in suppressing nematode’s immune system. It also led to the accumulation of ROS in the intestine of *C. elegans*.

Although ethanol could suppress the immune system of *C. elegans*, the nematode might sense it and elevate the expression of the ethanol dehydrogenase, *sodh-1*, as shown in the co-transcriptomic analysis. To characterize the role of ethanol dehydrogenase, *sodh-1* and *sodh-2*, gene knockouts and RNAi worms were tested in bioassay infected by *P. lavendulum*. The result showed that *sodh-1* mutant worms were more susceptible to fungal infection than N2 worms. Meanwhile, RNAi of *sodh-2* in *sodh-1* mutant was more susceptible to fungal infection than in *sodh-1* mutants. Together, these results indicated that ethanol dehydrogenases were required for the nematode to defend against fungal infection. Some other studies also revealed that ethanol dehydrogenases might be involved in animal immunity. For example, alcohol dehydrogenase was up-regulated in the hemolymph of *Drosophila* third-instar larvae 25 min after lipopolysaccharide (LPS) challenge (58). In *C. elegans* infected by *Staphylococcus aureus*, alcohol dehydrogenase *sodh-1* was also up-regulated by 12-folds (59). The *sodh-1* gene was also up-regulated when *C. elegans* was infected by a gram-positive bacteria, *Microbacterium nematophilum* (60). Most studies concerning *sodh-1* were conducted using omics methods, but the molecular mechanism in nematode innate immunity had not been studied previously.

Both N2 *C. elegans* and *sodh-1*-mutated nematodes were infected with *P. lavendulum*, but only ROS accumulation was detected in N2 *C. elegans*, indicating that the alcohol dehydrogenase plays an essential role in stimulating ROS production. Without fungal infection, nematodes were directly treated with ethanol, and it was found that there was a significant up-regulation of *sodh-1* and accumulation of ROS in the nematodes’ intestines. The exact pathway through which ROS are produced remains to be explored. From the transcriptomic analysis, we found many genes included in lipid metabolism-related pathways in the peroxisome, such as fatty acid beta-oxidation, unsaturated fatty acid beta-oxidation, other oxidation, and amino acid metabolism, were up-regulated. ROS may be a by-product of the active peroxisome, as previously reported (61).

**Fig 6:**
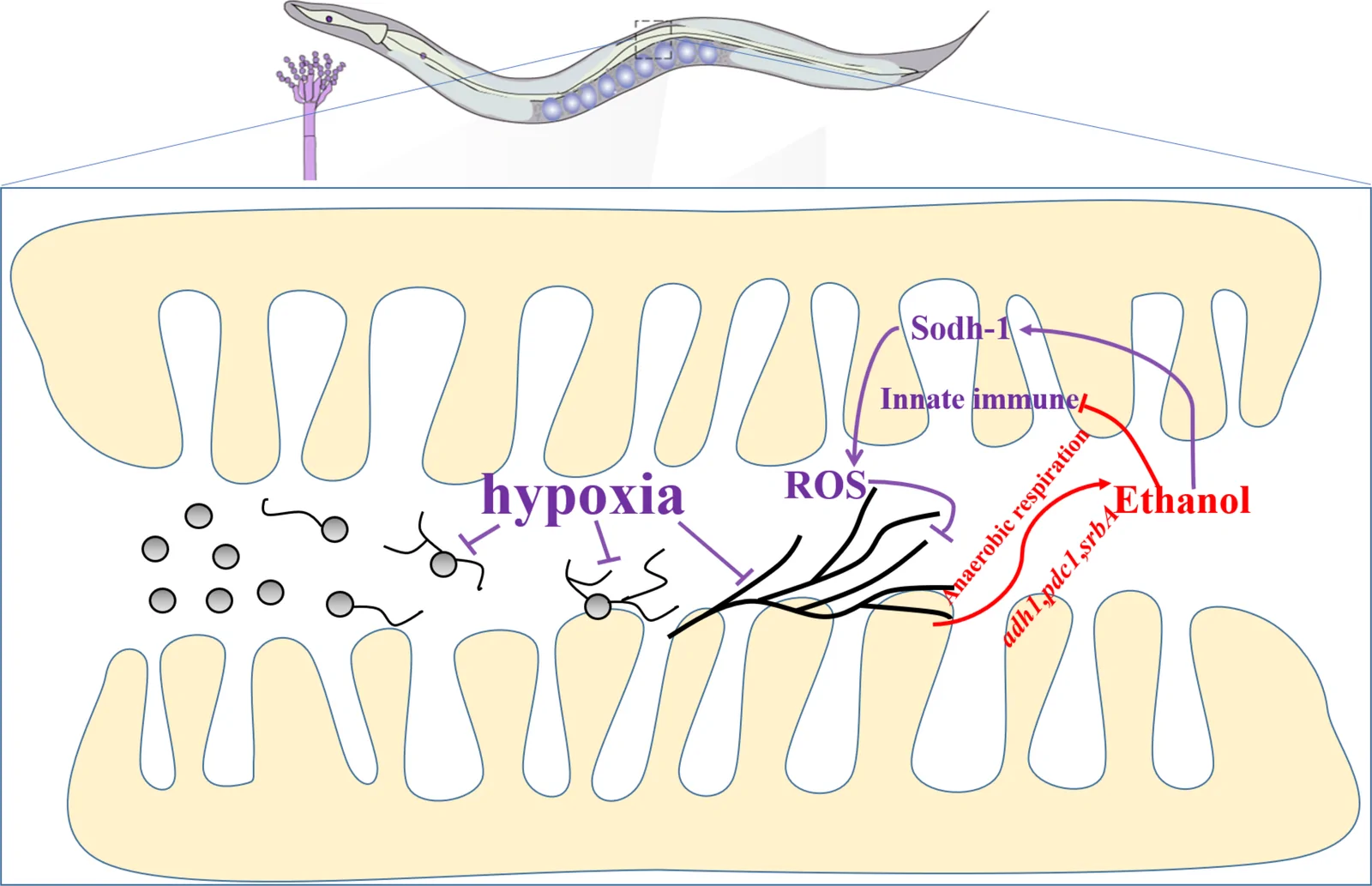
Model diagram of interactions between *P. lavendulum* and *C. elegans*. Purple, *C. elegans* actions; red, *P. lavendulum* actions.

In conclusion, we characterized a novel arms-race-like interaction mediated by ethanol, between the nematode *C. elegans* and its fungal pathogen *P. lavendulum* (Fig. 6). Free-living nematodes such as *C. elegans* mainly reside in humid temperate regions and reproduce in rotting fruits where microorganisms, especially the ethanol-producing yeasts, are concentrated (62 – 64). Ethanol may be sensed by nematodes commonly and thus affect the recognition of pathogenic microbes by nematodes. There are still many unanswered questions about this interaction. For example, how is ROS produced in the mycelia of *P. lavendulum*? Which receptor is responsible for ethanol sensing? Efforts are underway to resolve these and other issues.

## MATERIALS AND METHODS

### Fungal and nematode strains

*C. elegans* wild-type (N2）and *sodh-1* (tm2829) were all kindly provided by the Caenorhabditis Genetics Center (CGC; http://www.cbs.umn.edu/CGC), funded by NIH Office of Research Infrastructure Programs (P40 OD010440). *P. lavendulum* YMF1.00683 was isolated from the soil in Yunnan, China. RNAi bacterial strains were obtained from the Ahringer RNAi library (65).

### Hypoxia level detection in *C. elegans*

Young adult *C. elegans* (N2) were subjected to fungal infection with 1 × 10^8^ conidia of *P. lavendulum* for 5 days. Hypoxia level detection in *C. elegans* was performed using the Image-iT Green hypoxia agent (ThermoFisher Scientific), according to the manufacturer’s instructions (non-infected nematodes were used as controls).

### Gene knockout in *P. lavendulum*

Knockout of the genes *adh1*, *pdc1,* and *srbA* in PL followed the homologous-recombination strategy described previously (25). Homologous arms were PCR amplified using primers described in Table S1, and then inserted into the plasmid pPK2-SUR-GFP (kindly provided by Professor Weiguo Fang of Zhejiang University, China) (66), and transduced into PL via *Agrobacterium-tumefaciens*-mediated transformation and screened by PCR with primers described in Table S1.

### Nematode RNAi

RNAi bacterial strains containing targeting genes were obtained from the Ahringer RNAi library (67). The strains were fed to synchronized L1 larvae of *C. elegans* at 20°C. L4 RNAi larvae were used for bioassay (the HT115 strain was used as a control).

### ROS detection in *C. elegans*

To determine the accumulation of ROS under different conditions, the nematodes were strained with DHE or DCFH-DA and then observed under a Nikon Ni-U fluorescence microscope (Nikon, Japan). Briefly, nematodes were rinsed twice with M9 buffer, and then 250 µL of M9 and the corresponding staining agent (3 mM DHE or 25 µM DCFH-DA in the final concentration) were added, and the Eppendorf tube was wrapped in tin foil paper at 20°C for 2 h away from light. After straining, the dye was removed by centrifugation at 4,000 rpm for 2 min and rinsed with M9 for 3–4 times. The ROS levels of *C. elegans* were observed by fluorescence microscope and analyzed by Image-J software (National Institutes of Health), and statistical data were obtained using graphpadprism 9.

### Ethanol production detection by GC-MS

Ten microliters of conidia (1 × 10^8^ /mL in ddH_2_O) was inoculated into 10-mL MM liquid medium (68). The medium was placed in a sealed culture tank with a micro-aerobic airbag (Mitsubishi, Japan) to create a hypoxia environment. The control groups were cultured in a normal oxygen environment. All groups were shaken and cultured at 28°C, 180 rpm for 10 days.

The volatile compounds were extracted by headspace microextraction with an extraction needle (ACAR/PDMS/DVB) for 12 h. After extraction, the probe was put into the injection port of the GC-MS equipment (Agilent Technologies 7890A, 5975C). The initial temperature of the injection port is 250°C. After the probe was rotated out, the initial temperature of the column box was set at 40°C. After the analysis was completed, automatic integration was used, and the value of the integral parameter was 5.0. Finally, the data were retrieved, exported, and analyzed by comparing with the spectrum library of NIST11.L. Pure ethanol was added to fresh MM media for extraction and detection as a positive control.

### Real-time quantitative PCR

Total RNA was extracted as described previously (69). The methods of reverse transcription into cDNA and RT-qPCR were as previously mentioned (70). Actin was used as an internal reference gene in the experiment, and the primer sequences used are shown in Table S1.

### Transcriptome analysis

Young adult *C. elegans* (N2) were infected by 1 × 10^8^ conidia of *P. lavendulum* for 24 h or 5 days. Control samples were nematodes without infection and newly germinated mycelia of *P. lavendulum*. Total RNA of the samples was extracted and cDNA was synthesized for sequencing on the Illumina NovaSeq6000 platform. DEGs were screened by DESeq2. Then GO and KEGG enrichment were carried out by Goatools and KOBAS (71).

The L3 larvae were washed off from the nematode growth media (NGM) plates, and treated with 3 mM ethanol in M9 buffer on a rotating shaker for 8 h. The larvae were collected for RNA sequencing as described above. Nematodes cultured without ethanol were used as control. All samples were repeated three times.

### Bioassay

About 100 L4 *C*. *elegans* were added to NGM plates with *Escherichia coli* OP50 and 1 × 10^8^ *P. lavendulum* conidia (or mutant strains, no conidia added in the control group) and incubated at 20°C. Live nematodes were counted under a light microscope every 24 h. The adjusted mortality rate is calculated by the following formula: mortality_adjusted_ (%) =  100 ×  [(mortality_treated_ − mortality_control_)/(1 − mortality_control_)].

Mortality control means mortality of the group without fungal infection (feed with *E. coli* OP50).

### Statistical analysis

The statistical analysis was carried out mainly using GraphPad Prism 9 (GraphPad Software, Inc.). Nematode mortalities were analyzed with a one-way ANOVA. The growth rate and conidia production of the fungus were compared by *t*-test. The fluorescence intensity comparison of ROS strains was done by *t*-test.

## Data Availability

Raw transcriptome data are available in the Sequence Read Archive (SRA) with the BioProject accession number PRJNA885978.

## SUPPLEMENTAL MATERIAL

Fig.S1 to S7, Tables S1 to S3 spectrum.01270-23-s0001.pdf GO and KEGG enrichment analysis, survival rates of sodh-2 RNAi, NAC treatment,gene knockout of three genes, primers, and GC-MS composition analysis.

ASM does not own the copyrights to Supplemental Material that may be linked to, or accessed through, an article. The authors have granted ASM a non-exclusive, world-wide license to publish the Supplemental Material files. Please contact the corresponding author directly for reuse.
