# Supplementary material for: Ethanol mediates the interaction between Caenorhabditis elegans and the nematophagous fungus Purpureocillium lavendulum
Source: Microbiol Spectr. 2023 Aug 10;11(5):e01270-23. doi: 10.1128/spectrum.01270-23 (PMC10580998; doi:10.1128/spectrum.01270-23)
Supplement: Fig.S1 to S7, Tables S1 to S3 — GO and KEGG enrichment analysis, survival rates of sodh-2 RNAi, NAC treatment,gene knockout of three genes, primers, and GC-MS composition analysis. [file spectrum.01270-23-s0001.pdf]

Supplementary materials

Ethanol mediates the interaction between *C. elegans* and the nematophagous fungus *Purpureocillium lavendulum*

Xue-Mei Zhuang[1 ¶], Zhi-Yi Guo[1 ¶], Meng Zhang[1 ¶], Yong-Hong Chen[1], Feng-Na Qi[1], Ren-Qiao Wang[1], Ling Zhang[1], Pei-Ji Zhao[1], Chao-Jun Lu[1], Cheng-Gang Zou[1], Yi-Cheng Ma[1], Jianping Xu[1,2], Ke-Qin Zhang[1], Yan-Ru Cao[3]*, Lian-Ming Liang[1]*

[1] State Key Laboratory for Conservation and Utilization of Bio-Resources in Yunnan and The Key Laboratory for Southwest Microbial Diversity of the Ministry of Education, Yunnan University, Kunming, China.

[2] Department of Biology, McMaster University, Hamilton, Ontario, L8S 4K1, Canada.

[3] College of Agriculture and Life Sciences, Kunming University, Kunming, PR China.

¶These authors contributed equally to this work.

*Correspondence: lianglm@ynu.edu.cn (L-ML); yanrucao3@aliyun.com(Y-RC)

Fig. S1. GO and KEGG enrichment analysis of differently expressed genes (DEGs) between infected and uninfected *C. elegans*. (A) GO enrichment analysis of DEGs between 24 h infected and uninfected *C. elegans*. (B) KEGG enrichment analysis of DEGs between 24 h infected and uninfected *C. elegans*. (C) GO enrichment analysis of DEGs between 5-day infected and uninfected *C. elegans*. (B) KEGG enrichment analysis of DEGs between 5-day infected and uninfected *C. elegans*.

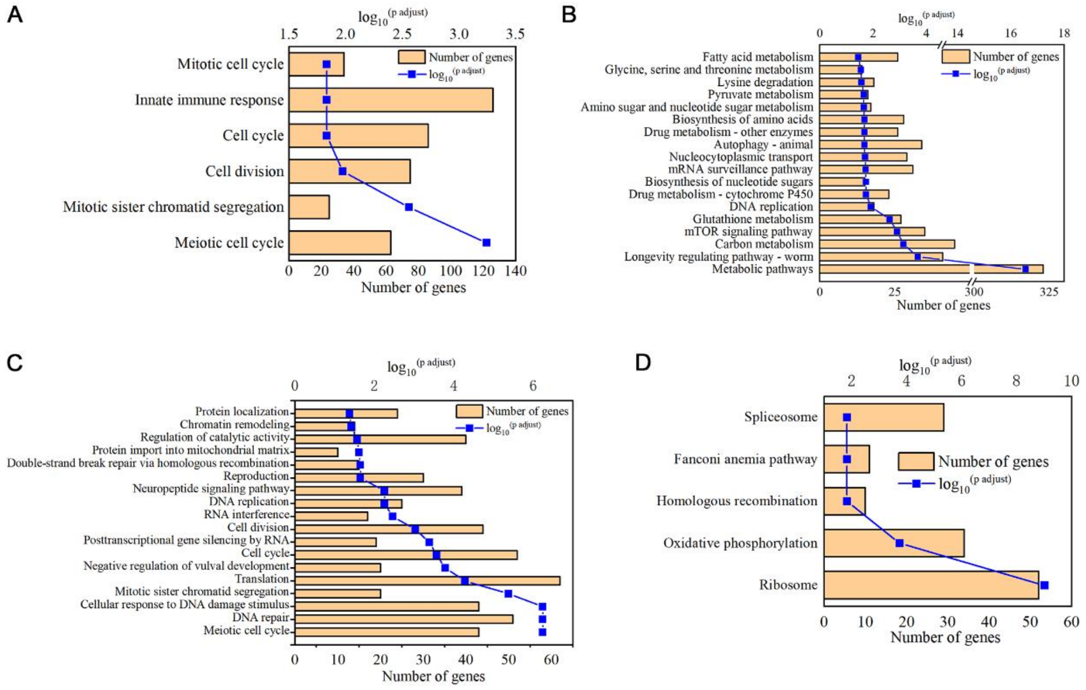

Fig.S2. GO enrichment analysis of DEGs in the fungus *P. lavendulum* between 5-day infected samples and pure culture-grown mycelia.

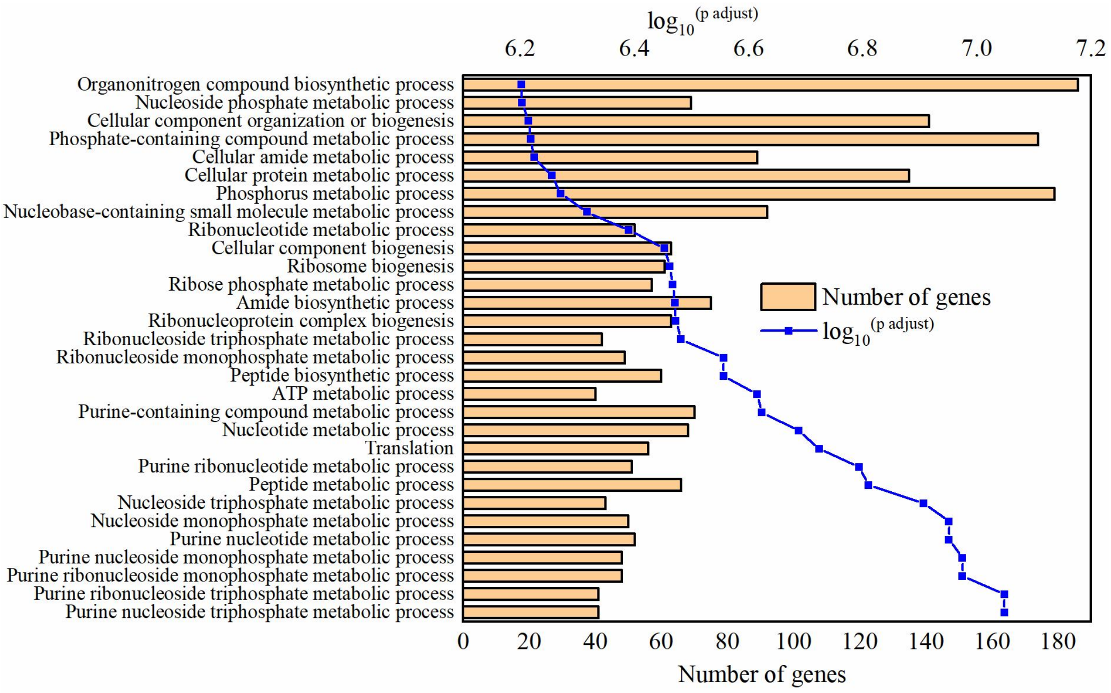

Fig. S3. Comparison of ajusted survival rates of *sodh-2* RNAi and EV of *C. elegans* exposed to *P. Lavendulum*.

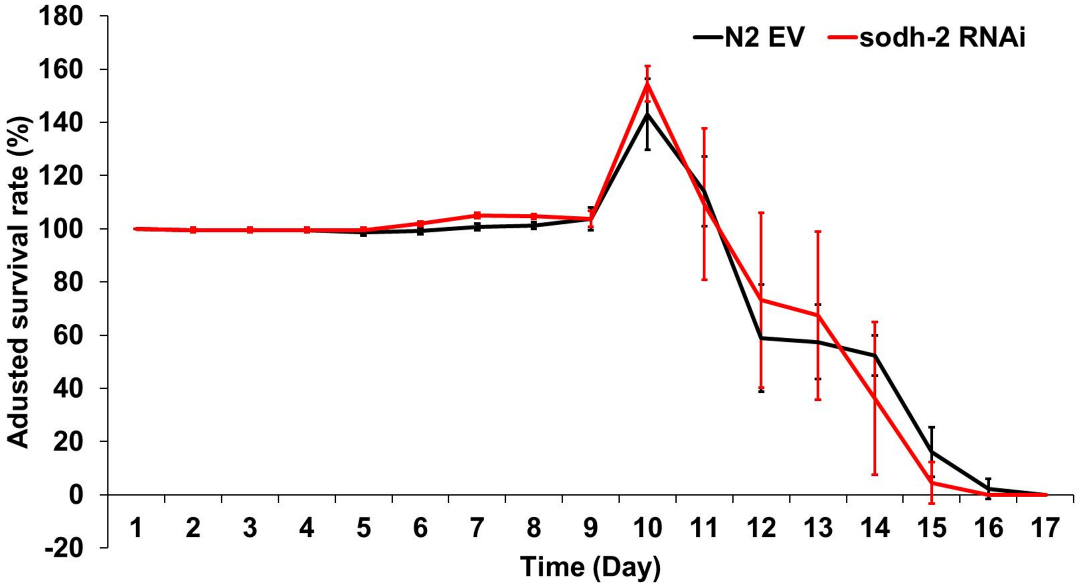

Fig. S4. NAC treatment affected conidia germination in the nematode intestinal tract and body-cavity leakage of nematodes when infected by *P. lavendulum*. (A) the different germination of the conidia in nematodes treated with NAC and not treated with NAC in 2day and 6 days after infection. (B) The body-cavity leakage of nematodes on day 2 and day 6 after infection was stained by food blue.

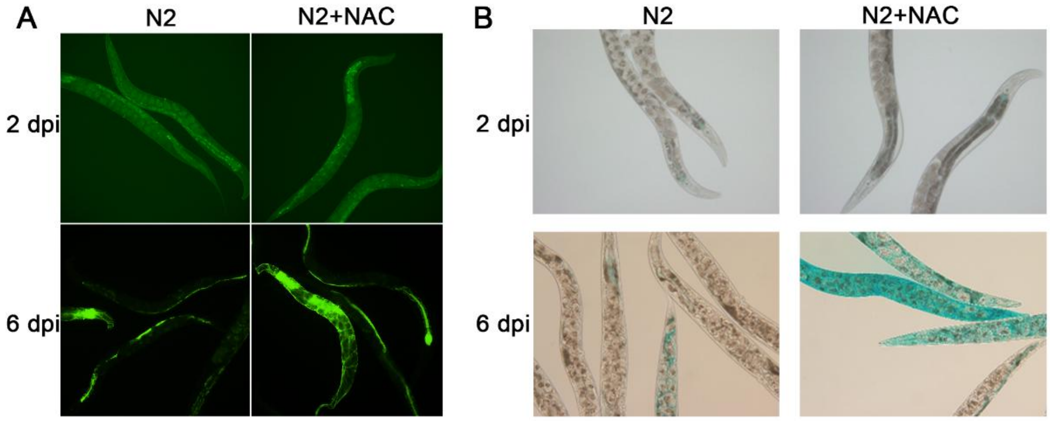

Fig. S5 gene knockout of three anaerobic respiration and ethanol synthases related genes. (A) Diagram of gene knockout. (B) PCR confirmation of *adh-1*. (C) PCR confirmation of *pdc-1*. (D) PCR confirmation of *srbA*.

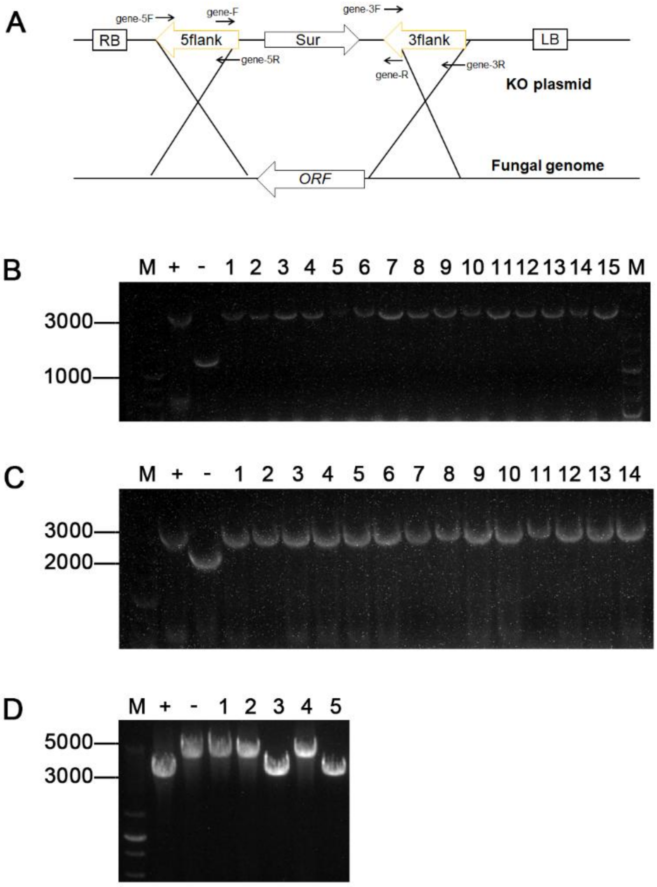

Fig. S6. GO enrichment analysis and KEGG enrichment analysis of up-regulated genes in nematode treated with ethanol

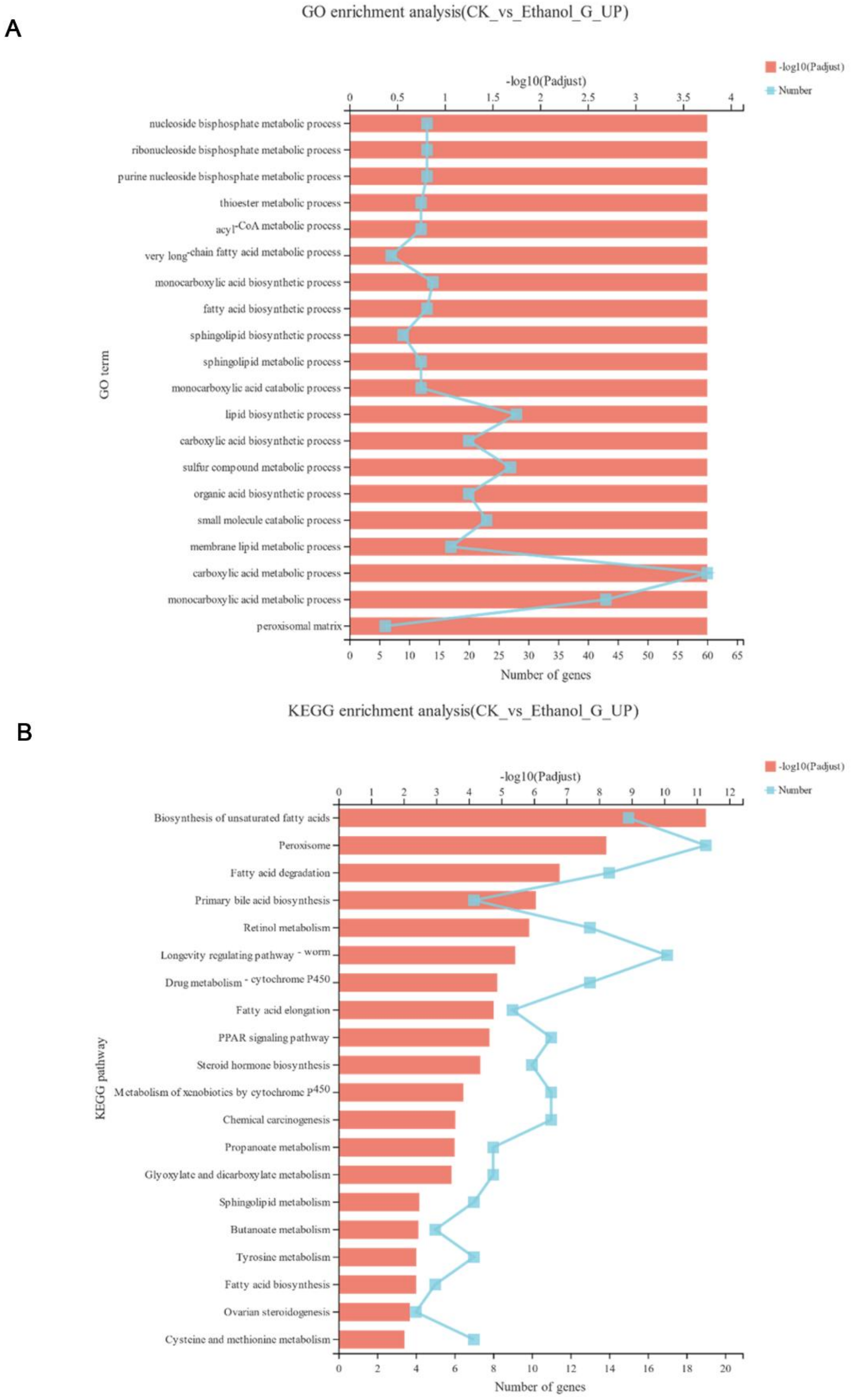

Fig. S7. GO enrichment analysis and KEGG enrichment analysis of down-regulated genes in nematode treated with ethanol

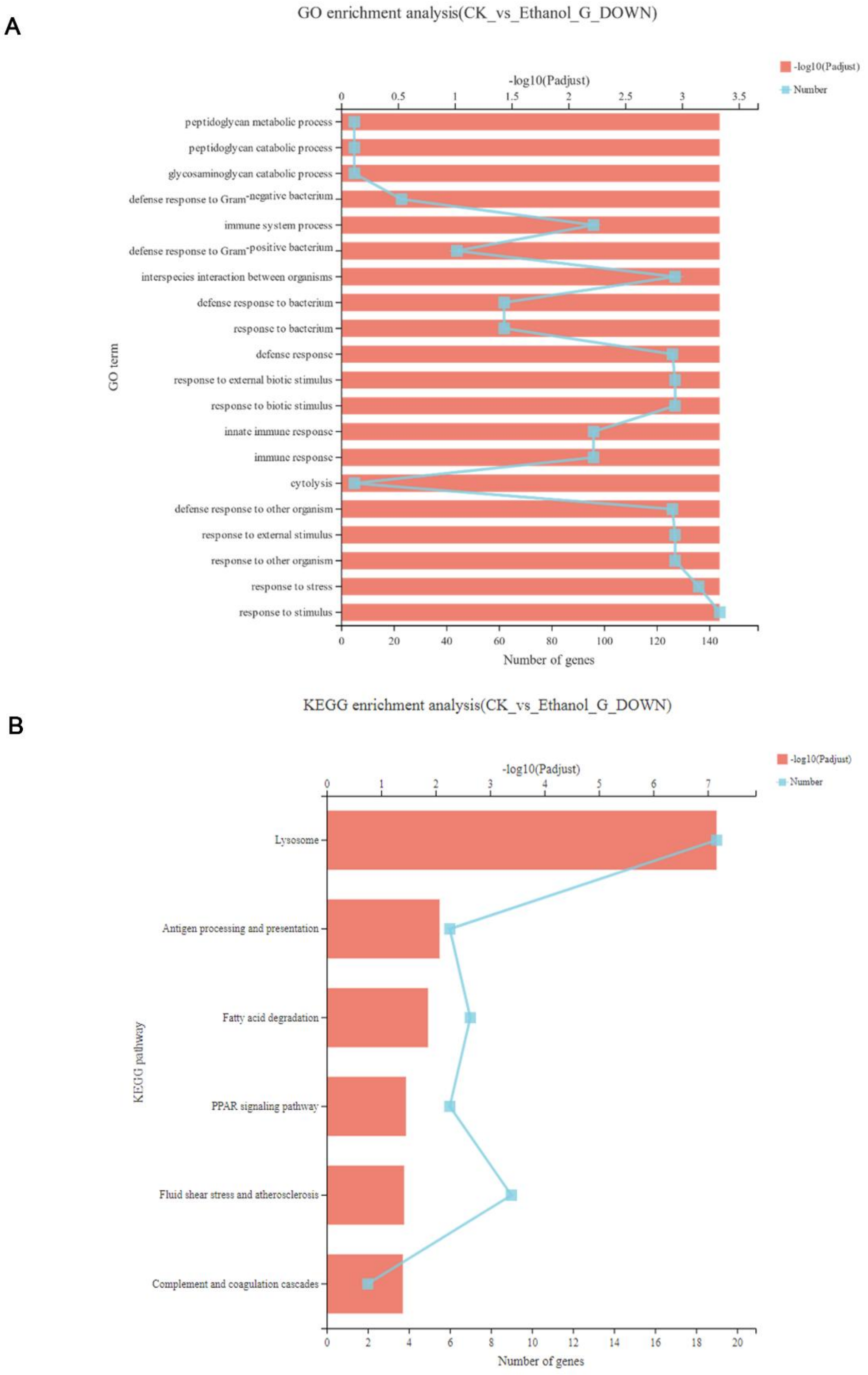

Table S1. primers used in this study

No	primer	Sequence（5→3′）	Function
1	adh1-5F	GGCCAGTGCCAAGCTTGCATGCCTGCA GGCTGTCGGGAGGCAGCACGCT	Amplify the upstream homologous fragment of *adh-1*
	adh1-5R	GGCGTTGGCACAGATCTAGTCTCTAGA CGTATCTAGAGGAGCAAGCA	
2	adh1-3F	ACGGGAATTGCATGCTCTCACACTAGTT GACGTCAATGAGTGCCGCT	Amplify the downstream homologous fragment of *adh-1*
	adh1-3R	TAATCGACCGACGGAATTGAGGATATC CCGTCTGCTTCCGTCGACGA	
3	pdc1-5F	GGCCAGTGCCAAGCTTGCATGCCTGCA GGCGTCCTTGATGGCCTCCAAT	Amplify the upstream homologous fragment of *pdc1*
	pdc1-5R	GGCGTTGGCACAGATCTAGTCTCTAGA GTACGGACGTCGCCGCACGG	
4	pdc1-3F	ACGGGAATTGCATGCTCTCACACTAGT GGAGAGTGTCTCTCTCGTCT	Amplify the downstream homologous fragment of *pdc1*
	pdc1-3R	TAATCGACCGACGGAATTGAGGATATC ACGGCGAACCTTGATTCTGT	
5	adh-1-F	CACCACCGCCCGTCTAAACACA	Verify that *adh-1* is knocked out
	adh-1-R	ACCCTTTTTCATCGTCCTCTGT	
6	pdc1-F	CACCACCGCCCGTCTAAACACA	Verify that the *pdc1* is knocked out
	pdc1-R	ACCCTTTTTCATCGTCCTCTGT	
7	srbA-5F	CCAAGCTTGCATGCCGTAAAGGCGTGA AGAAGAGGA	Amplify the upstream homologous fragment of *srbA*
	srbA-5R	AGATCTAGTCTCTAGTACGAGGCAGAT GGGGTGA	
8	srbA-3F	ATGCTCTCACACTAGTGTGCCTACGCCC TCTCTTC	Amplify the downstream homologous fragment of *srbA*
	srbA-3R	GACGGAATTGAGGATTCAACCCTACCG CTCCCTCT	
9	srbA-F	GCCCTCCCGCCTTTCACAAT	Verify that srbA is knocked out
	srbA-R	CTCCATCCACGGTCCACTTC	
10	sodh-1-F	TTCCAGTCCCATCTCCAGC	*sodh-1* real-time PCR primer
	sodh-1-R	TCCTTCGTGTCCTCCAACC	
11	Ce-actin-F	TCGGTATGGGACAGAAGGAC	*C. elegans* actin real-time PCR primer
	Ce-actin-R	CATCCCAGTTGGTGACGATA	
12	Adh-RT-F	GAGCCGTTGAGCCACTTGA	*adh1* real-time PCR primer
	Adh-RT-R	TACGCGGAGCATCCTGATT	
13	Pdc-RT-F	GCGATGTCGGAAGGATTGG	*pdc1* real-time PCR primer
	Pdc-RT-R	GATATGCCCGCGTGAACAA	
14	Srb-RT-F	AGGCTCGCCAACTCAATCA	*srbA* real-time PCR primer
	Srb-RT-R	GGTCGCATTCCTGCTCCAC	

15	Pl-actin-F	GAGGTAGTCGGTCAAGTCGC	*P. lavendulum* actin real-time PCR primer
	Pl-actin-R	TCCCATCAACCCCAAGTCC	

Table S2. GC-MS Composition Analysis Table (Normooxygen Condition)

PK	RT	Area Pct	Library/ID	Ref	CAS	Qual
1	1.5955	0.5641	(R)-(-)-2-Amino-1-propanol	903	035320-23-1	5
2	1.8897	0.3092	Carbon dioxide	81	000124-38-9	4
3	3.612	0.2487	Cyclotrisiloxane, hexamethyl-	79619	000541-05-9	72
4	4.8743	1.3295	Cyclotrisiloxane, hexamethyl-	79619	000541-05-9	90
5	7.7304	0.1831	Cyclotetrasiloxane, octamethyl-	141481	000556-67-2	87
6	7.9551	1.0655	Cyclotetrasiloxane, octamethyl-	141481	000556-67-2	83
7	9.8003	1.501	3-Octanone	12435	000106-68-3	91
8	9.9234	0.8983	3-Octanone	12435	000106-68-3	91
9	10.1854	3.1352	3-Octanone	12429	000106-68-3	59
10	11.881	0.8128	Cyclopentasiloxane, decamethyl-	196316	000541-02-6	90
11	14.7799	4.5155	1H-Indene, 1-ethylideneoctahydro-7a-methyl-, cis-	33797	056362-87-9	49
12	15.4431	0.3636	4-Methoxy-6-methyl-6,7-dihydro-4H-furo[3,2-c]pyran	37355	091894-15-4	35
13	15.5715	0.4403	3-Piperidinol, 1,4-dimethyl-, trans-	12994	037835-47-5	35
14	15.7801	0.4563	6-Tridecanone	59776	022026-12-6	43
15	16.1706	1.5145	Azulene	11936	000275-51-4	83
16	16.3096	1.3983	Cyclohexasiloxane, dodecamethyl-	225658	000540-97-6	91
17	16.5182	0.4473	2-Aminomethyl-5-methylamino-1,3,4-oxadiazole	12005	002937-92-0	49
18	16.6733	2.1061	1H-Cycloprop[e]azulene, 1a,2,3,4,4a,5,6,7b-octahydro-1,1,4,7-tetramethyl-, [1aR-(1a.alpha.,4.alpha.,4a.beta.,7b.alpha.)]-	64570	000489-40-7	70
19	17.3847	12.7462	Benzothiazole	15455	000095-16-9	91
20	17.465	1.9424	Benzothiazole	15455	000095-16-9	92

21	17.5452	11.3105	Benzothiazole	15455	000095-16-9	91
22	18.0105	5.6007	Benzothiazole	15455	000095-16-9	96
23	18.3207	1.6869	Benzothiazole	15455	000095-16-9	95
24	18.5614	4.9872	Benzothiazole	15455	000095-16-9	92
25	18.9305	6.1805	Quinoline, 1,2-dihydro-2,2,4-trimethyl-	40610	000147-47-7	60
26	19.1765	13.5809	Quinoline, 1,2-dihydro-2,2,4-trimethyl-	40610	000147-47-7	38
27	19.7969	3.2241	2-(1,3-Dimethyl-2,6-dioxo-1,2,3,6-tetrahydropurin-7-yl)-N-(6-methylpyridin-2-yl)acetamide	167092	1000322-24-0	35
28	20.1714	3.7528	Thieno[2,3-c]pyridine	15461	000272-12-8	47
29	20.8025	2.7018	2-Tridecanone	59780	000593-08-8	46
30	20.9309	0.5662	2-Undecanone	38224	000112-12-9	41
31	20.995	4.5007	Hexasiloxane, tetradecamethyl-	228692	000107-52-8	25
32	22.0327	0.5093	Hexasiloxane, 1,1,3,3,5,5,7,7,9,9,11,11-dodecamethyl-	222021	000995-82-4	43
33	22.1183	0.4015	Ethyl isopropyl dimethylphosphoramidate	57372	099520-56-6	38
34	22.3269	0.5626	Phenanthrene	44144	000085-01-8	72
35	22.3697	1.8277	Anthracene	44139	000120-12-7	78
36	22.9794	0.2598	Benzoic acid, 2,5-bis(trimethylsiloxy)-, trimethylsilyl ester	196461	003618-20-0	35
37	23.065	1.1333	1,1,1,5,7,7,7-Heptamethyl-3,3-bis(trimethylsiloxy)tetrasiloxane	225661	038147-00-1	50
38	23.9635	0.622	1,1,1,5,7,7,7-Heptamethyl-3,3-bis(trimethylsiloxy)tetrasiloxane	225661	038147-00-1	64
39	25.2419	0.2123	Sarcosine, N-isobutyryl-, tetradecyl ester	187376	1000321-27-5	38
40	26.3062	0.161	Propanamide	727	000079-05-0	47
41	27.023	0.2403	1,1,1,5,7,7,7-Heptamethyl-3,3-bis(trimethylsiloxy)tetrasiloxane	225661	038147-00-1	40

Table S3. GC-MS Composition Analysis Table (Hypoxia Condition)

PK	RT	Area Pct	Library/ID	Ref	CAS	Qual
1	1.1892	0.8432	Ethanol	94	000064-17-5	9
2	1.2908	2.6717	Ethanol	94	000064-17-5	40
3	1.5582	0.1941	Propanamide, 2-hydroxy-	2174	002043-43-8	42
4	1.5796	0.0486	Formaldehyde oxime trimer	15378	1000234-87-0	37
5	4.5588	6.2433	Cyclotrisiloxane, hexamethyl-	79617	000541-05-9	91
6	4.5856	5.9295	Cyclotrisiloxane, hexamethyl-	79619	000541-05-9	91
7	4.7139	9.2648	Cyclotrisiloxane, hexamethyl-	79619	000541-05-9	91
8	6.0618	6.6803	p-Xylene	5081	000106-42-3	97
9	6.388	0.8567	p-Xylene	5078	000106-42-3	95
10	6.618	2.1796	Styrene	4873	000100-42-5	95
11	6.6715	1.3474	Styrene	4873	000100-42-5	96
12	6.6983	0.9998	Styrene	4873	000100-42-5	95
13	6.7732	0.1653	Styrene	4873	000100-42-5	96
14	7.891	11.2322	Cyclotetrasiloxane, octamethyl-	141483	000556-67-2	83
15	9.0998	5.527	Oxime-, methoxy-phenyl-_	24837	1000222-86-6	76
16	9.1586	1.14	Oxime-, methoxy-phenyl-_	24837	1000222-86-6	70
17	9.2389	1.3971	Oxime-, methoxy-phenyl-_	24837	1000222-86-6	89
18	11.849	5.5353	Cyclopentasiloxane, decamethyl-	196318	000541-02-6	91
19	15.6359	0.6437	Ether, methyl 1-octadecenyl	129468	026537-06-4	55
20	16.3044	2.8967	Cyclohexasiloxane, dodecamethyl-	225657	000540-97-6	91
21	16.4007	1.0635	Eicosane	129491	000112-95-8	70
22	16.5986	3.9223	1H-3a,7-Methanoazulene, 2,3,6,7,8,8a-hexahydro-1,4,9,9-tetramethyl-, (1.alpha.,3a.alpha.,7.alpha.,8a.beta.)-	64538	000560-32-7	49
23	16.7537	0.2169	Benzothiazole	15455	000095-16-9	94
24	16.8607	0.9085	Benzothiazole	15455	000095-16-9	94
25	16.8928	0.3146	Benzothiazole	15455	000095-16-9	93
26	16.9356	0.2405	Benzothiazole	15455	000095-16-9	94
27	16.9676	0.1195	Benzothiazole	15455	000095-16-9	94
28	17.1067	0.7569	Benzothiazole	15455	000095-16-9	94
29	17.4704	1.0615	Benzothiazole	15455	000095-16-9	93
30	17.5132	0.3079	Benzothiazole	15455	000095-16-9	70
31	18.3155	2.1958	Pentasiloxane, dodecamethyl-	204092	000141-63-9	46
32	18.3904	1.8007	Eicosane	129490	000112-95-8	89
33	18.7274	1.6378	l-Alanine, N-neopentyloxycarbonyl-, undecyl ester	188661	1000322-69-6	30
34	18.845	5.3449	2,4,4-Trimethyl-3,4-dihydroquinoline	40607	063177-93-5	55

35	18.9306	2.997	1H-Indole, 2,3-dihydro-1,3,3-trimethyl-2-methylene-	40622	000118-12-7	42
36	19.2355	4.2457	Oxirane, [[4-(1,1-dimethylethyl)phenoxy]methyl]-	66017	003101-60-8	86
37	19.7971	1.8152	Silane, [[4-[1,2-bis[(trimethylsilyl)oxy]ethyl]-1,2-phenylene]bis(oxy)]bis[trimethyl-	228736	056114-62-6	50
38	20.9952	1.0537	Cyclononasiloxane, octadecamethyl-	242430	000556-71-8	52
39	22.0328	0.9422	Cyclononasiloxane, octadecamethyl-	242430	000556-71-8	68
40	22.9795	0.7813	Hexasiloxane, tetradecamethyl-	228692	000107-52-8	45
41	23.9637	0.8351	1,1,1,5,7,7,7-Heptamethyl-3,3-bis(trimethylsiloxy)tetrasiloxane	225661	038147-00-1	80
42	25.242	0.7496	Cyclononasiloxane, octadecamethyl-	242430	000556-71-8	72
43	27.0231	0.8927	Hexasiloxane, tetradecamethyl-	228692	000107-52-8	45
